# Preoperative imaging accuracy in size determination of prostate cancer in men undergoing radical prostatectomy for clinically localised disease

**DOI:** 10.1186/s13244-023-01450-5

**Published:** 2023-06-07

**Authors:** Wael Ageeli, Nabi Soha, Xinyu Zhang, Magdalena Szewcyk-Bieda, Jennifer Wilson, Chunhui Li, Ghulam Nabi

**Affiliations:** 1 Division of Imaging Sciences and Technology, School of Medicine, University of Dundee, Ninewells Hospital, Dundee, DD1 9SY UK; 2 Diagnostic Radiology Department, College of Applied Medical Sciences, Jazan University, Al Maarefah Rd, P.O. Box 114, Jazan, 45142 Saudi Arabia; 3 Division of Population Health and Genomics, School of Medicine, University of Dundee, Dundee, DD1 9SY UK; 4 Department of Clinical Radiology, Ninewells Hospital, Dundee, DD1 9SY UK; 5 Department of Pathology, Ninewells Hospital, Dundee, DD1 9SY UK; 6 School of Science and Engineering, University of Dundee, Dundee, DD1 4HN UK

**Keywords:** Cancer, Size, Multi parametric MRI, Prostate, 3D printing

## Abstract

**Objectives:**

To compare the accuracy of pre-surgical prostate size measurements using mpMRI and USWE with imaging-based 3D-printed patient-specific whole-mount moulds facilitated histopathology, and to assess whether size assessment varies between clinically significant and non-significant cancerous lesions including their locations in different zones of the prostate.

**Methods:**

The study population included 202 men with clinically localised prostate cancer opting for radical surgery derived from two prospective studies. Protocol-based imaging data was used for measurement of size of prostate cancer in clinically localised disease using MRI (*N* = 106; USWE (*N* = 96). Forty-eight men overlapped between two studies and formed the validation cohort. The primary outcome of this study was to assess the accuracy of pre-surgical prostate cancerous size measurements using mpMRI and USWE with imaging-based 3D-printed patient-specific whole-mount moulds facilitated histopathology as a reference standard. Independent-samples *T*-tests were used for the continuous variables and a nonparametric Mann–Whitney *U* test for independent samples was applied to examine the distribution and median differences between mpMRI and USWE groups.

**Results:**

A significant number of men had underestimation of prostate cancer using both mpMRI (82.1%; 87/106) and USWE (64.6%; 62/96). On average, tumour size was underestimated by a median size of 7 mm in mpMRI, and 1 mm in USWE. There were 327 cancerous lesions (153 with mpMRI and 174 for USWE). mpMRI and USWE underestimated the majority of cancerous lesions (108/153; 70.6%) and (88/174; 50.6%), respectively. Validation cohort data confirmed these findings MRI had a nearly 20% higher underestimation rate than USWE (*χ*^2^ (1, *N* = 327) = 13.580, *p* = 0.001); especially in the mid and apical level of the gland. Clinically non-significant cancers were underestimated in significantly higher numbers in comparison to clinically significant cancers.

**Conclusions:**

Size measurement of prostate cancers on preoperative imaging utilising maximum linear extent technique, underestimated the extent of cancer. Further research is needed to confirm our observations using different sequences, methods and approaches for cancer size measurement.

## Key points

- The study assessed size of prostate cancer on MRI and ultrasound shear wave elastography (USWE) and compared with size on microscopic examination (histopathology) after radical prostatectomy.
- In comparison to histopathology, there was significant underestimation of prostate cancer size by imaging using MRI and USWE.
- Specific message is for men opting for focal treatment of prostate cancer

## Introduction

Prostate cancer size assessment with preoperative imaging is crucial for the staging of disease [1]. Size of the primary tumour is also a major prognostic indicator and is one of the three parameters used in the American Joint Committee on Cancer/Union for International Cancer Control (AJCC/UICC) cancer staging; although T does not represent the precise size of the tumour, this does indicate the location and extent of the tumour in the prostate gland. The size of cancers seen on imaging is not only used for staging but also for risk stratification, particularly in localised prostate cancer [2]. Mathematical modelling and survival data in other sites show cancers displaying a direct correlation between the size of cancer and its lethality, irrespective of the methods of detection.

In prostate cancer, preoperative size assessment is achieved by Digital Rectal Examination (DRE), ultrasonography or MR imaging. In general, the ability of DRE and B mode transrectal ultrasonography to quantify the size of cancerous lesions remains poor, however, with the introduction of ultrasound shear wave elastography (USWE), measurement of cancerous lesions has become possible and the interest in the role of ultrasound has been further explored [3]. The accuracy of mpMRI for the determination of tumour size using PI-RADS classification has been reported by various methods in different studies, citing a range of degrees of correlation between mpMRI and histopathology [4–6].

There are, however, a few issues with the reported literature on this topic. Firstly, the studies failed to account for the consideration that conventional prostate gland histopathology sectioning with the posterior side down, the cutting plane may not match the imaging plane [4]. Moreover, imaging coils, surgical resection and histopathology tissue processing can deform the prostate’s shape and cancerous lesions [5, 6], even when using image analysis software [7, 8]. The variability in the sectioning of radical prostate specimens can be confounded with the histopathological location relative to imaging modalities [6, 9]. Thus, whole-mount pathology slides may show distinct depths angles and shapes of the prostate much different to clinical images. In order to improve registration accuracy, guides or templates were used to obtain uniform sections or slices [9, 10], but these methods do not ensure the correct orientation of the specimens. Secondly, the reported results for size determination on imaging are mixed. Some research shows that mpMRI gives an overestimation of tumour size [11, 12], whereas others showed that mpMRI underestimated the actual tumour size [13–15]. Almost all previous studies have defined the relationship between mpMRI and pathology as reliant on imprecise methods, such as volume approximation manual registration, and two-dimensional measurements.

To improve the accuracy and address the issues highlighted above, prostate specimens were sliced using imaging-based, 3D-printed patient-specific whole-mount moulds for each participant in the present study. The moulds were used to hold the prostate in the same orientation and shape observed in the images. The sections were then analysed by a uropathologist and histopathology was used as a reference standard.

The aims of this study were:

1. To compare the accuracy of pre-surgical prostate size measurements using mpMRI and USWE with imaging-based 3D-printed patient-specific whole-mount moulds facilitated histopathology.
2. To assess whether size assessment varies between clinically significant and non-significant cancerous lesions including their locations in different zones of the prostate.

## Materials and methods

### Study population

**Fig. 1:**
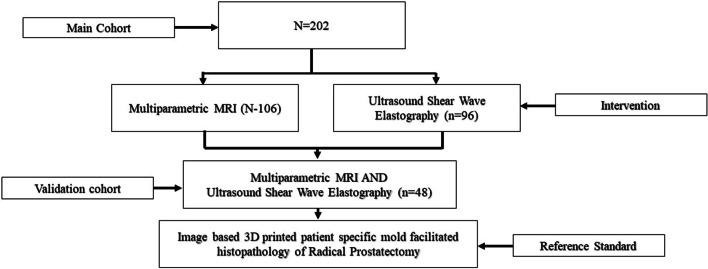
Study illustration

The study analysed images acquired during protocol-based two prospective studies between 2013 and 2018 [16, 17]. In the first study Wei et al. [16] prospectively recruited men for transrectal ultrasound shear wave elastography, and showed good diagnostic accuracy for prostate cancer detection. In the second study, Magdalena et al. prospectively [17] assessed the role of pre-biopsy mpMRI and the benefits of US/MRI image fusion guided biopsies. The studies had ethical approval through the East of Scotland Ethical committee and Caldicott permission (IGTCAL5626) to access the healthcare follow-up data specifically for this study [18]. In the study, 202 men with available data on imaging-based 3D-printed patient-specific whole-mount moulds-based histopathological processing were analysed. There were 106 men with pre-surgical multiparametric mpMRI and 96 men with pre-surgical USWE imaging data. Men with both mpMRI and USWE in the same patient (*n* = 48) formed a validation cohort. The histopathology of each participating man was reviewed by an experienced uro-pathologist (J.W.). Patients with confirmed PCa on TRUS guided biopsies, coupled with availability of both or one of pre-surgical USWE and mpMRI, and the diagnosis confirmed by radical prostatectomy were included. Patients were excluded if whole amount pathology images, both or either of imaging modalities (USWE images, mpMRI images) were unavailable or patients with prior radiotherapy, transurethral resection of the prostate and hormonal therapy. Table 1 shows patients characteristics. The study design is graphically illustrated in Fig. 1. The primary outcome of this study was to assess the accuracy of pre-surgical prostate cancerous size measurements using mpMRI and USWE with imaging-based 3D-printed patient-specific whole-mount moulds facilitated histopathology as a reference standard.

**Table 1:** Patient characteristics in the radical prostatectomy (*n* = 202)

Patients (N)	202
Age (LRP year)	
Median (IQR*)	67.0 (64–71)
PSA level (ng/mL)	
Median (IQR)	9.4 (7.3–13.5)
Prostate Weight (mL)	
Median (IQR)	60.0 (47.1–78.5)
PSAD (ng/mL^2^)	
Median (IQR)	0.2 (0.2–0.1)
Gleason score	*n* (%)
3 + 3	7 (3.0)
3 + 4	101 (48.0)
4 + 3	35 (17.0)
3 + 5	21 (10.0)
4 + 4	3 (1.0)
4 + 5 or more	45 (21.0)
pT stage	*n* (%)
pT2a	11 (5.0)
pT2b	3 (1.0)
pT2c	106 (50.0)
pT3a	67 (32.0)
pT3b	23 (11.0)
pT4	2 (1.0)
Lymph node status	*n* (%)
pN0	187 (88.0)
pN1	13 (6.0)
pNX	12 (6.0)
PIRADS	*n* (%)
3	16 (7.6)
4	59 (27.8)
5	137 (64.6)

*IQR, interquartile range; ^PSA, prostatic specific antigen; &PSAD, prostatic specific antigen density

The secondary objective was to assess whether the location or clinical significance of cancerous lesions had any impact on size differences between imaging and histopathology.

Tumour size measurements of the prostate lesions were carried out by 2 radiologists with at least 5 years’ experience. Any discrepancies between the two were resolved by consensus. Following a literature review of several previous studies that reported measurement differences between cancers on imaging and histopathology, the difference in lesions size of ≤ 1 mm was considered as concordant [19–23]. The lesion was considered overestimated or underestimated in both imaging modalities if the size differences of the tumour on imaging were > 1 mm larger or smaller than the pathologic lesion size, respectively. We used the same size definition for both mpMRI and USWE.

### Measurement of cancerous lesions

**Fig. 2:**
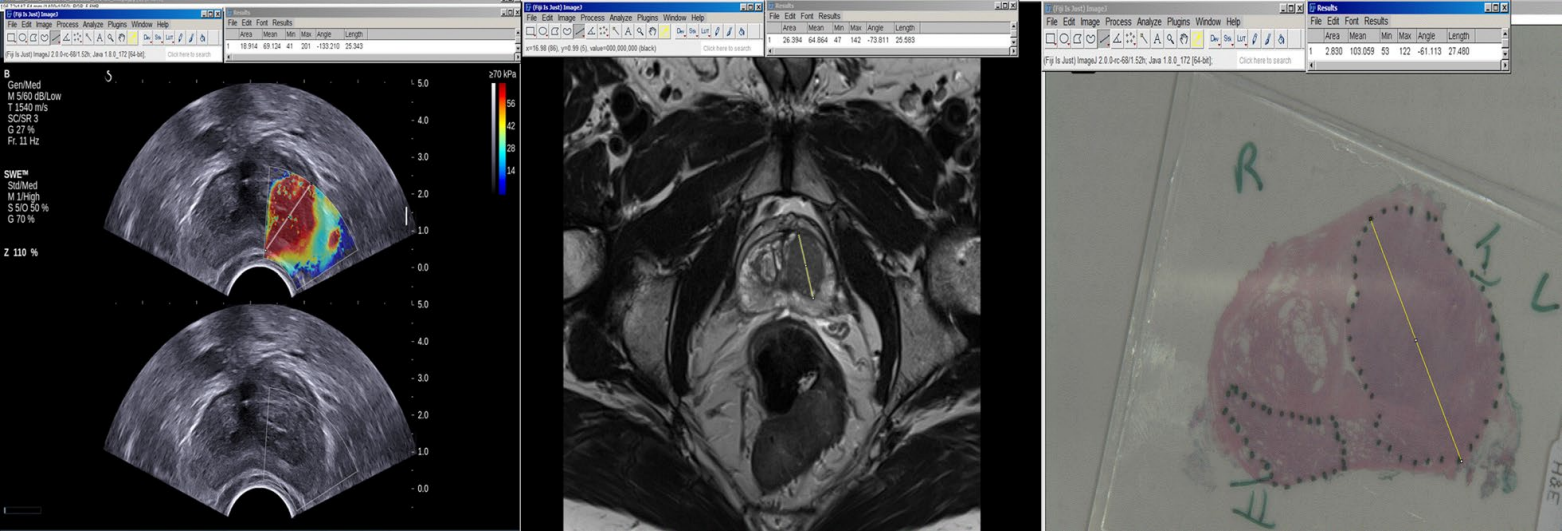
An example of Images obtained from a 74 years old man with a PSA level of 9 ng/mL. The patient underwent mpMRI and USWE examinations. Final diagnosis was prostate cancer with Gleason score 4 + 5

The longest diameter of malignant prostate lesions (maximum linear extent) seen and classified as PI-RADS 3 or more on T2W MRI or seen in the colour map of USWE was measured (Fig. 2). A similar method was used for histopathological lesions seen in the prostate sections using imaging-based 3D-printed patient-specific whole-mount moulds. Comparison of size and accuracy detection of tumours between the two imaging modalities was carried out in different locations of prostate gland from base to apex.

### Magnetic resonance imaging protocols

mpMRI scans were performed for each patient with 3 T scanners (TIM Trio, Siemens, Erlangen, Germany) 6–8 weeks after the biopsy. The mpMRI protocol for prostate cancer was obtained from the 2012 European Society of Uro-radiology Guidelines (ESUR) [24]. The scan protocol includes (T1 weighted image (T1WI), T2 weighted image (T2WI), diffusion-weighted image (DWI), apparent diffusion coefficient (ADC) and dynamic contrast-enhanced (DCE)). The scanning protocol was previously described and the endorectal coil was not used [25, 26]. The protocol combines anatomical sequences (TSE T2 and T1WI) with functional imaging, which includes DWI sequences with three b-values (0, 400, and 1000 s/mm^2^) and a separate high b-value (2000 s/mm^2^) acquisition, as well as dynamic contrast-enhanced (DCE) sequences (3D fast gradient-echo sequences with temporal resolution of 4 s, using 2 mL/kg of gadolinium-based contrast agent. The longest diameter (maximum linear extent) was measured using the T2WI sequence. Two qualified and experienced uro-radiologists (S.M.B., J.S.) analysed and achieved a consensus for all the MR images and were blinded to the histopathology data.

### Ultrasound shear wave elastography protocol

The USWE technique measures the shear wave speed produced by specialised ultrasound transducer in the target tissues. A dynamic map of tissue stiffness (representative of Young Modulus of elasticity) is created reflecting different speeds of shear waves tissue areas in real-time. Detailed technology of imaging is described elsewhere [27, 28]. All USWE images were obtained using a transrectal endocavitory transducer (SuperSonic Imagine, Aix en Provence, France) with patients either in lateral or lithotomy position. USWE mode was applied and elastograms of the prostate were acquired from cranial to caudal direction for each prostate lobe. The stiff regions were coloured red, while the soft and elastic regions were coloured blue. Stiff We calculated the mean elasticity of each target zone using the ultrasound machine's software version of the Young module. The USWE images were taken from base to apex in transverse planes with a gap of 4 to 6 mm. The most suspected planes containing cancer were labelled and rebuilt offline into 3-D images. Rotating transducer in different directions to scan suspicious cancer regions ensured verification of abnormalities and accurate measurement of their dimensions. The ratio between abnormal and normal areas and three stiffness measurements of Shear wave speed in m/s or Young’s modulus in kPa using pseudo-colour map were recorded by three researchers (G.N., C.W. and D.U.) independently.

### Histopathology protocol

Imaging-based 3D-printed patient-specific whole-mount moulds were designed using imaging data and printed according to our published protocol [25]. Briefly, pre-surgical T2-weighted images (T2WI) images of prostate in three planar views (axial, coronal and sagittal) were obtained using 3 T MRI machine. The slice thickness of each slice was 3 mm with 0.6 mm gap and the scan resolution for axial view of 0.63 × 0.63 mm^2^. After a detailed analysis of 2D pelvic images moulds were created using MIMICS software (Medical Image Segmentation for Engineering on Anatomy), stereolithography (STL) files. The border of the prostate capsule was identified using an expert uro-radiologist help and a detailed review of 2D pelvic imaging, and the prostate was segmented in one direction (mainly axial) and changed in the other two directions. The moulds held the prostate in the same shape and orientation as seen on the mpMRI. The 3D mold included a computer-generated sequence of parallel slits uniformly spaced, each corresponding to a recognised slice of T2-weighted MRI. Following surgery, prostate specimens were sliced in the axial orientation from base to apex immediately by using a multi-bladed slicing tool [4]. The steps followed were: first, segmentation of MRI data in biomedical software MIMICS, second, mold fabrication in CAD software SolidWorks (Innova systems, Cambridge), third, 3D printout from rapid prototyping machine MakerBot (Nottingham, UK), fourth, post-radical prostatectomy specimen before dyeing and mold placement, fifth, slicing of prostate specimen with a single blade, sixth, sliced sections shown in the mould and lastly, specimen slices arranged from apex to base. Histopathology was reviewed by two uro-pathologists, one at the time of initial reporting and other during the multidisciplinary team discussions.

### Data analysis

The participants were divided into mpMRI and USWE groups. The validation cohort (*n* = 48) had both imaging modalities. Patient’s age (in years), prostate-specific antigen (PSA), prostate volume, and prostatic specific antigen density (PSAD) in the two groups were measured and the values were compared to assess any discrepancy in patient characteristics between the mpMRI and USWE groups. The continuous data of mpMRI and USWE groups were first tested for normal distribution by the Kolmogorov–Smirnov Test of Normality. The mean (m) and standard deviation (SD) were described if the variable followed a normal distribution. The median (M) and interquartile range (IQR) were presented if the variable was not normally distributed. Independent-samples *T*-tests were used to compare the means of the continuous variables that were normally distributed. Otherwise, a nonparametric Mann–Whitney *U* test for independent samples was applied to examine the distribution and median differences between mpMRI and USWE groups. A cross-tabulation was carried out in order to compare the proportions of size underestimation of prostate cancer between mpMRI and USWE [29]. Underestimation is clinically important for treating cancer, so the study focused on the underestimation size rate of cancer between the imaging modalities. Pearson Chi-square, degree of freedom (df) and *p* value were calculated and presented. The total number of lesions were counted based on zones. Statistical analyses were conducted by SPSS V23.0.

In this study, Gleason scores 3 + 3 and 3 + 4 were considered as low/intermediate significant prostate cancer. Following University College London (UCL 2) definition, Gleason score ≥ 4 + 3 was considered to be highly significant prostate cancer. Bland–Altman plots were performed to demonstrate the level of agreement in mpMRI vs prostatectomy histopathology and in USWE vs prostatectomy histopathology. In order to prevent the effect of non-normally distributed differences (mpMRI-pathology, and USWE-pathology), logarithmic transformation was applied in the measurement of tumour size in mpMRI, USWE and prostatectomy histology [30]. The natural log average of the image-based and histopathological measurement of tumour size was plotted against the natural log difference between the two measurements for both modalities. Mean of log difference, upper and lower agreement limits with their confidence intervals were presented in the Bland Altman plots. The Bonferroni adjustment, which adjusted *p* value by times of the tests, was used to account for multiple testing. Adjusted *p* value equal to 0.05/ times of tests. The alpha level was set at 0.05/ times of tests to determine two-tailed significance.

## Results

### Patient population and characterisations

The patient’s age in the mpMRI group was normally distributed with mean and SD of 67.3 ± 5.7 years, respectively. Age in the USWE group, prostate-specific antigen (PSA), prostate volume and Prostate-specific antigen density (PSAD) in both the groups did not follow a normal distribution and therefore the Mann–Whitney *U* test was applied for comparing patient characteristics and results as shown in Table 2. There were no statistically significant differences between patients in the mpMRI and the USWE group in their age, PSA level, prostate volume or PSAD.

**Table 2:** Characteristics of patients in mpMRI and USWE group

Patient characteristics	MRI group (*n* = 106) Median (IQR*)		SWE group (*n* = 96) Median (IQR)		Mann–Whitney U	Z-Score	p
Age in years	67.5	(7.3)	70	(8.0)	4276.0	−1.96	0.051
PSA^^^ Level (ng/mL)	9.9	(4.0)	9.9	(6.7)	4913.5	−0.42	0.674
Prostate volume (mm)	60.3	(29.5)	60.0	(35.4)	5082.0	−0.01	0.992
PSAD^&^	0.16	(0.10)	0.16	(0.13)	4825.0	−0.63	0.529

*IQR, interquartile range; ^^^ PSA, prostatic specific antigen; ^&^PSAD, prostatic specific antigen density

Several (*n* = 13) Chi-square tests were conducted to compare the underestimation of prostate cancer between mpMRI and USWE. The tests were run on the overall number of patients and lesions; lesions on base, mid and apex levels; patients with significant and non-significant cancer. Patients whose cancer was located in the transition zone, in the peripheral zone and both transition and peripheral zone; and finally lesions of prostate cancer that located in the transition zone, in the peripheral zone and both transition and peripheral zone. After Bonferroni adjustment, the adjusted statistically significant *p* value is 0.0038 (0.05/13).

### Calculation of underestimation in tumour size in MRI and SWE group

The mpMRI imaging and radical prostatectomy histopathological lesions size were not normally distributed. A Mann–Whitney *U* test showed that there was a statistically significant difference (*U* = 8439.5, *p* < 0.001) between the prostate cancer lesion size measured by mpMRI compared to prostatectomy. The median lesion size measured by mpMRI was 16 mm compared to 23 mm via prostatectomy suggesting that the mpMRI underestimated the measurement of the cancer lesions.

The USWE imaging and prostatectomy pathological lesions sizes were not normally distributed. A Mann–Whitney *U* test showed that there was a statistically significant difference (*U* = 14,119, *p* < 0.001). The median lesion size measured by USWE was 20 mm compared to 21 mm via prostatectomy suggesting that the USWE is underestimating the measurement of cancer lesions.

### Comparing measurement difference between mpMRI Group and USWE Group

The median of lesion size difference in the mpMRI group (lesion size measured by mpMRI- lesion size measured after prostatectomy) and in the USWE group (lesion size measured by USWE- lesion size measured after prostatectomy) were −5 mm and −1 mm, respectively. Neither of the difference was normally distributed. Mann–Whitney *U* test showed that there was a statistically significant difference (*U* = 10,099.5, *p* < 0.001) between the difference in prostate cancer lesion size measured by mpMRI and USWE. This indicates that USWE measured prostate cancer lesions more accurately than mpMRI.

**Fig. 3:**
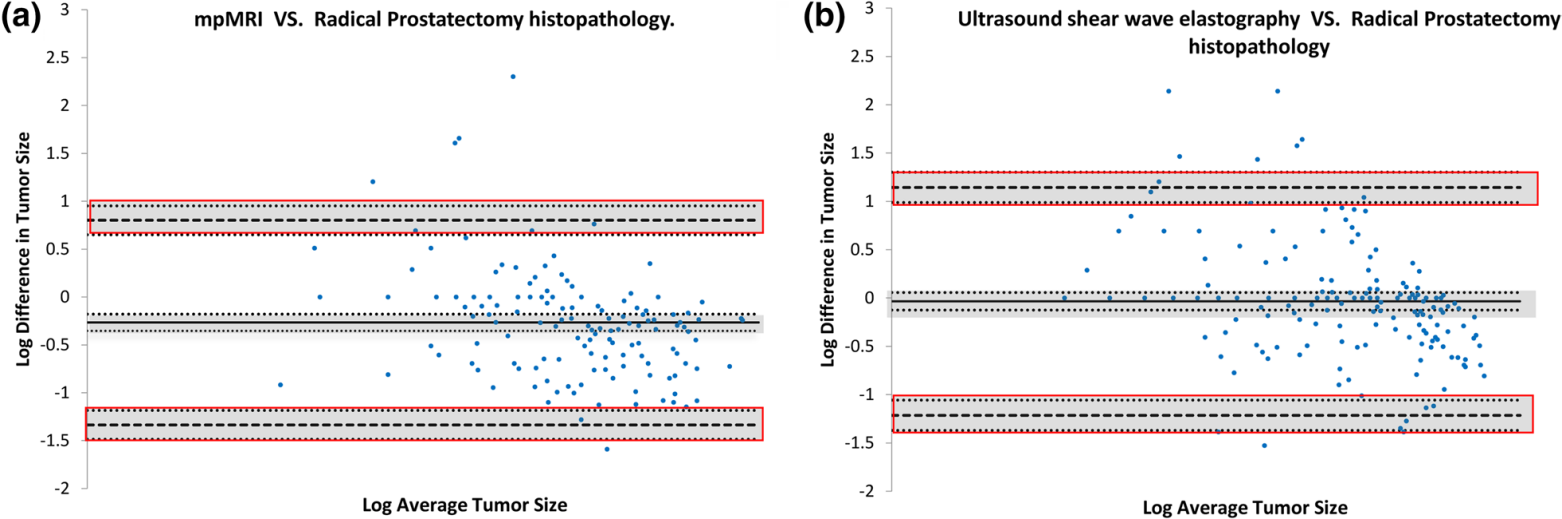
Bland–Altman plots for mpMRI vs prostatectomy (*n* = 153) (**a**) and USWE vs prostatectomy (*n* = 174) (**b**) with logarithmic transformation. The y-axis represents the natural log differences between the each modality and the histopthalogy. The x-axis represents the log average of each modalities with the histopathology. The solid black line in the centre represents the mean log difference, the dashed black lines indicate the upper and lower limits of agreements (mean ± 1.96 SD), and the shaded areas are confidence interval limits of mean and agreement limits

Figure 3 shows Bland–Altman plots with disagreement between measurements using imaging and histopathology. In Fig. 3a, the log mean difference of tumour lesion size via mpMRI and radical prostatectomy was −0.2670 (95% CI, −0.3540 to −0.1798). The line of equality (which is 0) was not within the confidence interval of the log mean difference. Whereas in Fig. 3b, the log mean difference of tumour lesion size via USWE and prostatectomy was −0.0347 (95% CI, −0.1247 to 0.0553). The mean of log difference between USWE and prostatectomy was not statistically significantly different from 0.

### Accuracy of size measurements of mpMRI and USWE with histopathology

**Fig. 4:**
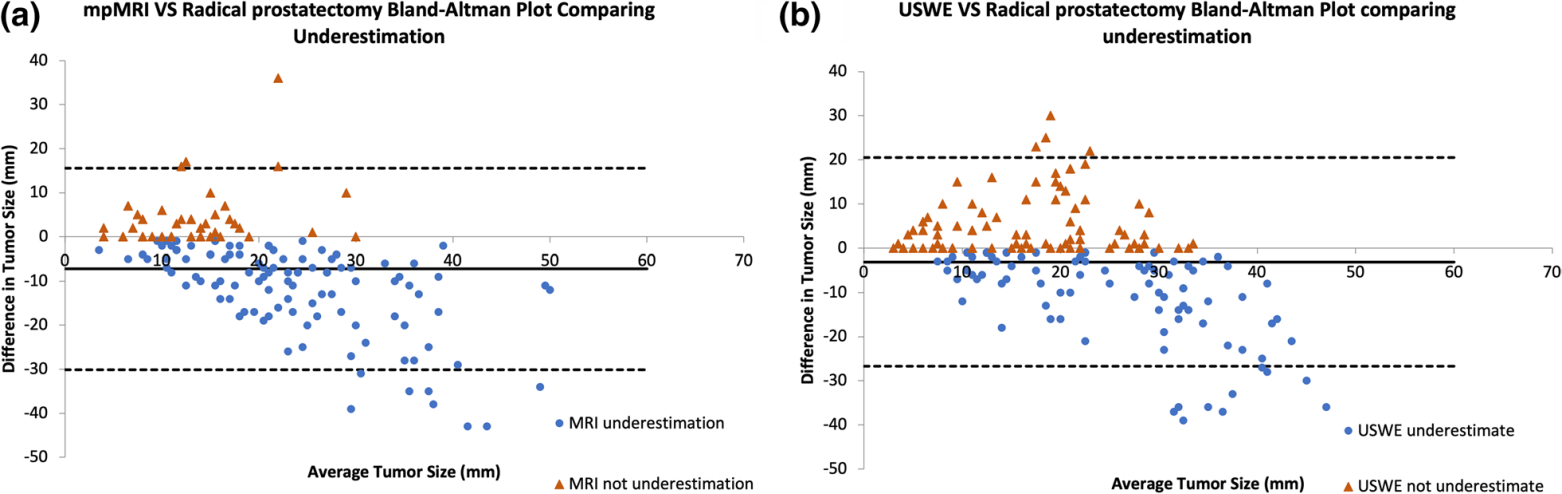
Bland–Altman plots for mpMRI (**a**) and USWE (**b**). The y-axis represents the differences between the each modality and the histopathology. The x-axis represents average of each modalities with the histopathology. The solid black line in the centre represents the mean difference, the dashed black lines indicate the upper and lower limits of agreements (mean ± 1.96 SD)

Table 3 shows that a significant number of men (82.1%; 87/106) had an underestimation of prostate cancer using mpMRI or USWE (64.6%; 62/96). In terms of cancerous lesions-based analyses, the proportions of underestimation were 70.6% and 50.6%, for mpMRI and USWE respectively. A Chi-square test of independence was performed to examine the relation between mpMRI and USWE in the underestimation of prostate cancer lesions. The relation between these variables was statistically significant, *χ*^2^ (1, *N* = 327) = 13.580, *p* = 0.0002. mpMRI had a 20% higher underestimation rate compared to the USWE test for prostate cancer lesions (Fig. 4).

**Table 3:** Distribution of underestimation in prostate cancer using mpMRI and USWE among all patients and lesions

Measurements	Underestimation	%	Not underestimation	%	Pearson Chi-square	Degree of freedom	*p* value*
Total number of patients							
MRI (*n* = 106)	87	82.1	19	17.9	7.964	1	0.0048
SWE (*n* = 96)	62	64.6	34	35.4			
Total number of lesions							
MRI (*n* = 153)	108	70.6	45	29.4	13.580	1	0.0002
SWE (*n* = 174)	88	50.6	86	49.4			

*MRI* magnetic resonance imaging, *SWE* shear wave elastography

*To make the result statistically significant, the adjusted *p* value for comparison is 0.0038

### Accuracy of size measurements of mpMRI and USWE with histopathology according to the zonal location of prostate cancerous lesions

If the lesions were located at the mid-level of the prostate, using the mpMRI test would have a 76.0% (92/121) underestimation rate while the rate dropped to 56.5% (78/138) using USWE. The Chi-square test of independence showed the result was statistically significant, *χ*^2^ (1, *N* = 259) = 10.882, *p* = 0.0010. If the lesions were located at the apex level of the prostate, using the mpMRI test would have a 74.3% (84/113) underestimation rate while the rate dropped to 56.0% (75/134) using USWE imaging. The Chi-square test of independence showed the result was statistically significant, *χ*^2^ (1, *N* = 247) = 9.017, *p* = 0.0027. mpMRI had a nearly 20% higher underestimation rate than USWE in prostate lesions located in the mid and apex level as presented in Table 4. In terms of zonal location of prostate cancerous lesions, there was a higher underestimation using mpMRI in comparison to USWE, however, no statistically significant differences were observed for size estimation between the zones as shown in Tables 5 and 6.

**Table 4:** Distribution of underestimation in prostate cancer using mpMRI and USWE in lesions located at base, mid and apex level

Measurements	Underestimation	%	No underestimation	%	Pearson Chi-square	Degree of freedom	*p* value*
Lesions located at base level							
MRI (*n* = 59)	50	84.7	9	15.3	3.637	1	0.0565
SWE (*n* = 64)	45	70.3	19	29.7			
Lesions located at mid level							
MRI (*n* = 121)	92	76.0	29	24.0	10.882	1	0.0010
SWE (*n* = 138)	78	56.5	60	43.5			
Lesions located at apex level							
MRI (*n* = 113)	84	74.3	29	25.7	9.017	1	0.0027
SWE (*n* = 134)	75	56.0	59	44.0			

*MRI* magnetic resonance imaging, *SWE* shear wave elastography

*To make the result statistically significant, the adjusted *p* value for comparison is 0.0038

**Table 5:** Distribution of underestimation in prostate cancer using mpMRI and USWE among patients whose cancer was located in transition zone, in peripheral zone and in both transition and peripheral zone

Measurements	Underestimation	%	Not underestimation	%	Pearson Chi-square	Degree of freedom	*p* value*
Patient’s cancer in transition zone							
MRI (*n* = 13)	11	84.6	2	15.4	11.589	1	0.0007
SWE (*n* = 9)	1	11.1	8	88.9			
Patient’s cancer in peripheral zone							
MRI (*n* = 45)	31	68.9	14	31.1	0.535	1	0.4646
SWE (*n* = 36)	22	61.1	14	38.9			
Patient’s cancer in both zones							
MRI (*n* = 48)	45	93.8	3	6.2	5.743	1	0.0166
SWE (*n* = 51)	39	76.5	12	23.5			

*MRI* magnetic resonance imaging, *SWE* shear wave elastography

*To make the result statistically significant, the adjusted *p* value for comparison is 0.0038

**Table 6:** Distribution of underestimation in prostate cancer using mpMRI and USWE lesions of prostate cancer that located in transition zone, in peripheral zone and in both transition and peripheral zone

Measurements	Underestimate prostate cancer	%	Not underestimate prostate cancer	%	Pearson Chi-square	Degree of freedom	*p* value*
Lesions located in transition zone							
MRI (*n* = 20)	14	70.0	6	30.0	7.216	1	0.0072
SWE (*n* = 29)	9	31.0	20	69.0			
Lesions located in peripheral zone							
MRI (*n* = 85)	49	57.6	36	42.4	3.887	1	0.0487
SWE (*n* = 89)	38	42.7	51	57.3			
Lesions located in both zones							
MRI (*n* = 48)	45	93.8	3	6.2	7.616	1	0.0058
SWE (*n* = 56)	41	73.2	15	26.8			

*To make the result statistically significant, the adjusted *p* value for comparison is 0.0038

Finally, we assessed differences between clinically significant and clinically non-significant cancers. Generally, there was an underestimation of clinically significant prostate cancer size by both mpMRI and USWE, however, mpMRI had a higher underestimation by 26.2% compared to USWE for clinically non-significant cancerous lesions and the results were statistically significant, *χ*^2^ (1, *N* = 107) = 8.307, *p* = 0.0039 as presented in Table 7. Clinically significant prostate cancers were underestimated by median size of 1.74 mm and clinically non-significant cancers were underestimated by median size of 2.96 mm using mpMRI and for USWE the size underestimation for clinically significant cancer was a median size of 1.60 mm and for non-significant cancer,it was a median size of 2.79 mm.

**Table 7:** Distribution of underestimation in prostate cancer using mpMRI and USWE among patients with significant and non-significant cancer

Measurements	Underestimation	%	Not underestimation	%	Pearson Chi-square	Degree of freedom	*p* value*
Patients with significant cancer							
MRI (*n* = 51)	43	84.3	8	15.7	0.762	1	0.3825
SWE (*n* = 44)	34	77.3	10	22.7			
Patients with non-significant cancer							
MRI (*n* = 55)	44	80.0	11	20.0	8.307	1	0.0039
SWE (*n* = 52)	28	53.8	24	46.2			

*MRI* magnetic resonance imaging, *SWE* shear wave elastography

*To make the result statistically significant, the adjusted *p* value for comparison is 0.0038

In the validation cohort, again, only a small number of lesions (3/73; 4.1%) were seen on mpMRI accurately matched to histopathological size. mpMRI overestimated approximately one in five lesions (17/73; 23.2%). Whereas in the majority of the lesions (53/73; 72.6%) mpMRI underestimated the size of the cancer lesions in the prostate. Similar to mpMRI, USWE underestimated just over half the lesions (43/73; 58.9%) and its performance in accurate matching and overestimation of the size was (1/73; 1.3%) and (29/73; 39.7%) respectively. The average size of the tumour underestimated by mpMRI and USWE was 4.8 mm and 1.3 mm, respectively, and the median was 3.4 (0.3–3.4) mm and 0.9 (0.3–16.4) mm, respectively.

## Discussion

### Key findings of the study

The purpose of our study was to determine the accuracy of USWE and mpMRI for predicting the size of prostate cancer using imaging-based 3D-printed patient-specific whole-mount moulds guided histopathology as a reference standard. The study confirms that pre-biopsy mpMRI and USWE significantly underestimated the size of prostate cancer; albeit less with USWE. A higher discrepancy was observed for mpMRI in clinically non-significant cancers. There were significant differences in size estimation of cancer foci located in different levels of the prostate gland.

A consensus on establishing reliable criteria for the measurement of tumour size is crucial for determining treatment options and monitoring treatment responses. Uniform criteria for documenting the size of cancers and reporting response, recurrence, and disease-free interval, as well as the grading of acute and subacute toxicity in solid tumour therapy, were proposed in 1979 [31].

In the present study, most cancer foci were underestimated using mpMRI as well as USWE despite application of the Bonferroni adjustment. This approach successfully reduced the false-positive results caused by multi-testing. The underestimation rate for the mpMRI test was higher in comparison to USWE for cancers in the mid and apical level of the gland, non-significant cancers (26.2%), and lesions located in both transition zone and peripheral zone (20.6%). Clinically non-significant cancers were underestimated by mpMRI more than significant prostate cancers and this may have implications for active surveillance. There are several possible reasons to explain this observation. Firstly, it is well-known that low grade tumours are under estimated by MRI due to less cellularity and no neovascularity [32]. Secondly, hazy appearance of clinically non-significantly cancers especially at margins on mpMRI might not be obvious and more likely to be missed from measurements. Diffuse infiltration of cancer cells with a lower percentages of cancer cells from tumour core to normal prostate becomes difficult to detect on imaging. There is no ideal imaging modality which has potential to provide what is so-called tumour purity at the edges.

USWE size estimation performed better, however, this modality is not commonly used in clinical practice. In view of this, findings from this study become important for future research in USWE and healthcare practice. Moreover, findings should also be taken into account to facilitate planning in the treatment of prostate cancer foci where margins of treatment need to be significantly wider than the region of interest. The size underestimation may be expected for several reasons: prostate cancer heterogeneity; mpMRI evaluation cbeing difficult in the apex given the small size of this region and its location at the margin of the prostate [33]; some focal lesions might be unnoticed on standard DWI protocols due to signals received from surrounding benign prostatic tissue which overshadows the lesion [34] and the fuzzy appearance of cancer margins on mpMRI may cause the reader to overestimate the less visible smaller lesions while underestimating larger ones. Furthermore, small prostate tumours can be crescentic in shape as well as subcapsular in location. Because of the low T2 signal strength and crescentic form of the surrounding capsule, these tumours can be difficult to identify on traditional T2-weighted imaging. A wedge-shaped area of decreased T2 signal intensity and decreased apparent diffusion coefficient (ADC) within the PZ is typically detected at the posterior midline of the prostate base. The specific reason for this benign feature is unknown, however, it may be connected to the fusion of the prostate capsule and overlaying fascia by the junction of the two lobes [35]. The deep location of the TZ and CZ may be a factor of the USWE in the underestimation the lesion size. Furthermore, the transition zone frequently contains calcifications, making PCa differentiation difficult. Moreover, when benign prostate hyperplasia progressed, the transitional zone and core zone grew relatively deep; SWE technology limits the penetrated depth of shear wave pulse in tissue to 3 to 4 cm; correct SWE data in the anterior prostate could not be collected if the volume of the prostate was large. According to Jonmarker et al., formalin injection has no influence on tissue shrinkage as determined on microscopic slides and so has no effect on prostate cancer volume calculation [36].

Bland–Altman plots in Fig. 3 answered the question of whether using the same or identical method of measurements for different modalities- histology and imaging would show any agreement or correlation. Traditionally, this method is used for measurements using two modalities with objectives that one might replace the other with sufficient accuracy for the intended purpose of measurement [37]. When compared, two Bland–Altman plots with logarithmic transformation, the line of equality (which means the perfect agreement which is 0) is not in the confidence interval of mean difference in the mpMRI group, which indicates there is a significant difference, i.e. mpMRI constantly underestimates the tumour size compared to prostatectomy histopathology. In the USWE group, the line of equality is within the confidence interval of mean difference and there is no show of statistically significant difference. Measurement in USWE and radical prostatectomy histopathology with logarithmic transformation agree with one another, which in a way proved that USWE performed better in tumour size measurements compared to mpMRI. The confidence intervals of mean difference and of the agreement limits describe a possible error in the estimates due to a sampling error [30].

### Comparison of findings to the reported literature

Based on the reports in the literature, 5 to 10 mm margin around the region of interest (ROI) is sufficient for focal treatment of prostate cancer [38, 39]. However, our results indicate that tumour size was underestimated by more than 10 mm in a third of lesions on mpMRI, and around one-fifth of lesions were underestimated by more than 10 mm in USWE. Turkbey et al. [13] observed an underestimation of mean index volume of 0.16 cc (7%) and correlation coefficient of 0.63. Even though the strength of the correlation was comparable to that in our study in the two groups, the ROIs in the Tukbey et al. [13] study was larger and more closely matched the volume of the tumours. However, inconsistent use of 3D moulds and the ellipsoid formula which does not account for the actual tumour shape limited the accuracy of the tumour volume. Our findings for mpMRI are in agreement with the data reported by Priester et al. [4]. Isebeat et al. reported that there was a significant correlation between tumour volume measures at histology and tumour volumes determined by T2w and DW imaging. The DCE MR image-based tumour volume measurements revealed no significant correlation [40].

Farrokh et al. [1] recently reported data that proves that USWE is much more precise for measuring cancer size. Another study by Farrokh et al. [41] confirmed that USWE can predict the lesion size precisely compared to other modalities.

Some underestimation may be expected because of tumour heterogeneity and blending with the surrounding healthy tissue [42]. This can be mitigated by steps aimed at improving the accuracy of tumour contours such as image processing software to inform radiologists about which regions are most likely to contain cancer, and dimensions can be adjusted if needed [43, 44]. Also, targeted biopsy systems can confirm whether cancer exists along multiple vectors in the prostate gland. Extra millimetres of margins of ROI should be treated during focal prostate therapy [45].

Sang et al. [46] reported that the sensitivity and specificity of USWE in detecting prostate cancer is high, and it can distinguish between malignant and benign prostate lesions. Samuel Borofosky et al. [47] reported that prostate cancerous lesions can be missed or their size can be underestimated by mpMRI. Overall, at least one significant tumour was either underestimated in size or missed in 31 (31%) of 100 patients. Also, Rosenkrantz et al. [42] found that mpMR imaging had a sensitivity of 76% when compared to matched pathology specimens. Similarly, Le et al. [48] reported a sensitivity of 47% for the detection of all lesions in MRI. Moreover, almost 30% of more than 7 Gleason grade tumours and > 1 cm in size were missed at imaging. However, Leddy et al. reported that MRI significantly overestimated tumour size in breast cancer (68.4%) compared with mammography (33.3%) and ultrasonography (45.6%) [49]. Onesti et al. [50] found a significant overestimation of tumour size on MRI, particularly in tumours measuring > 2.0 cm. Ahmed et al. reported sensitivity and specificity of 90% and 88% respectively for men with prostate cancer and PSA < 20 ng/mL using USWE in comparison to 93% and 93% for those with PSA > 20 ng/mL [51]. However, Zippel et al. and Ko et al. reported that ultrasound shear wave elastography overestimated breast cancer size [52, 53]. In summary, our observations add knowledge to the current literature of size estimation of localised prostate cancer on imaging, specifically using preoperative USWE.

The current study has some limitations. Our study only included patients treated with radical prostatectomy with pre-surgical mpMRI and USWE imaging. Therefore, generalizability to patients opting for non-surgical treatments remains unknown. This was a single-institution study, and our results need further external validation. Future studies could be focused on 3D size measuring of cancerous lesions for both the modalities and comparison with histopathology. A recognizable limitation of the study is that we only measured the difference in size of one dimension, and did not address the measurements made to ROI areas (2 dimensions) and volumetric segmentation (3 dimensions) in both histopathology and imaging.

There are several strengths of the study which include prospectively protocol-based imaging data, use of robust reference standard and statistical analyses as well as confirmation of findings using an internal validation cohort. We have also analysed data based on the clinical significance of cancers which may have a higher implication for future clinical practice.

### Implications for future research

Accurate tumour size measurement is an important prognostic indicator for prostate cancer and interventionists rely on the radiological estimate to guide complete treatment of the disease. Multifocal disease, such as prostate cancer makes the issue challenging and worth further research. Certainly, future research should focus on intraoperative imaging methods to detect occult diseases such as bioimpedance spectroscopy, better contrast agents and the use of biopsies to ensure completeness of therapy.

Findings from the present study where underestimation of prostate cancer size using imaging is seen should be taken into scoring or risk stratifications of localised disease in the future. The addition of information on underestimation may make the PI-RADS scoring system a better prognostic test for cancer survivorship.

## Conclusions

Preoperative imaging using mpMRI significantly underestimated the size of prostate cancer in men undergoing radical prostatectomy in comparison to USWE. The study confirms that clinically non-significant cancers are more underestimated than significant ones using anatomical registration based on imaging-derived 3D-printed patient-specific whole-mount moulds facilitated histopathology as a reference standard.

## Data Availability

The datasets generated during and/or analysed during the current study are available from the corresponding author on reasonable request.
